# SuPAR - A future prognostic biomarker in emergency medicine

**DOI:** 10.1186/1757-7241-23-S1-A31

**Published:** 2015-07-16

**Authors:** Rebecca M Østervig, Lars Køber, Jakob L Forberg, Lars S Rasmussen, Jesper Eugen-Olsen, Kasper Iversen

**Affiliations:** 1 Department of Anaesthesia, Centre of Head and Orthopaedics, Rigshospitalet, University of Copenhagen, Copenhagen, Denmark; 2 Department of Cardiology, Rigshospitalet, University of Copenhagen, Copenhagen, Denmark; 3 Clinical Research Centre, Hvidovre Hospital, University of Copenhagen, Copenhagen, Denmark; 4 Department of Emergency Medicine, Hospital Of Northern Zealand, Hillerød Hospital, Hillerød, Denmark; 5 Department of Cardiology, Herlev Hospital, University of Copenhagen, Copenhagen, Denmark

## Background

Efficient triage in the Emergency Departments (ED) is important to identify patients in need of urgent care. Biomarker measurements may aid these clinical decisions. suPAR, soluble urokinase-type plasminogen activator receptor, is a non-specific biomarker reflecting inflammation and is a strong prognostic marker for several diseases. This study investigated suPAR's predictive capacity to identify high- and low-risk patients in the Emergency Department.

## Method

This study was part of a prospective cohort study carried out at Hillerød University Hospital (TRIAGE-study). The prognostic value of suPAR was compared to the prognostic value of triage category based on the information from the systematic triage tool, Danish Emergency Process Triage (DEPT) in prediction of 30-days mortality. Blood samples were taken upon arrival to the ED. Patients admitted to the ED from September 2013 to December 2013 were included in the study. suPAR levels were measured in EDTA-plasma using the CE/IVD approved suPARnostic ELISA (ViroGates, Denmark).

## Results

Serum was available for analysis of suPAR in 5,992 patients (94% of the admitted patients). Mean age was 59.8 years and 50.1% were female. The mean concentration of suPAR was 5.5 ng/ml (± 3.6) and there was a significant correlation between suPAR level, CRP level (R^2 ^= 0.09), and leucocyte count (R^2 ^= 0.02), p < 0.01 for both. Mortality at 30 days was 3.6%. ROC analyses of the prognostic value of suPAR in relation to 30-day mortality showed that the area under the curve (AUC) was 0.85 (95% CI 0.82-0.87), similar analyses of the triage category showed an AUC of 0.62 (95% c.i. 0.58-0.66). Cox regression analysis of 30-day mortality in relation to suPAR quartiles showed that the hazard ratio for the second quartile was 2.2, third quartile 6.5, and highest quartile 38.4 (p < 0.001). In a multivariable analysis including gender, age, CRP, leucocyte count, and triage category, suPAR remained an independent predictor of 30-day mortality with a hazard ratio for second quartile of 4.5, third quartile 8.3 and highest quartile 26.9.

## Conclusion

In unselected patients admitted to an Emergency Department, suPAR is an independent marker of short-term mortality. suPAR could potentially help clinicians in the initial risk assessment of acutely admitted patients.

